# The Occurrence of Depression Among Adults With Sickle Cell Disease in Saudi Arabia

**DOI:** 10.7759/cureus.44595

**Published:** 2023-09-03

**Authors:** Muazzam M Sheriff, Alhanouf K Alsharif, Fai A Almalki, Wed A Alqurashi, Dhyy A Alqurashi, Hanin H Abusabah, Reem A Alshanbari, Fatimah J Alshalab, Heba B Sindi, Tasneem A Bahrawi, Alya Z Alqurashi, Youssof Al Omar, Samaher G Basalib

**Affiliations:** 1 Microbiology and Immunology, Ibn Sina National College for Medical Studies, Jeddah, SAU; 2 Medicine and Surgery, Ibn Sina National College for Medical Studies, Jeddah, SAU; 3 Medicine, Ibn Sina National College for Medical Studies, Jeddah, SAU

**Keywords:** public health, hematology, psychiatry, occurrence in adults, complications, saudi arabia, depression, sickle cell disease

## Abstract

Introduction

Sickle cell disease (SCD) is recognized as a widely prevalent genetic disorder that impacts individuals globally and is inherited within families. The primary cause of SCD is a singular genetic mutation that affects the globin chain of the hemoglobin protein. Depression and its symptoms are frequently observed in individuals with SCD. This observation has led to a higher probability of adverse health outcomes. Therefore, the primary objective of this study was to analyze depression among Saudi Arabian adults with sickle cell disease.

Method

The online survey questionnaire was administered in both Arabic and English to collect essential information regarding SCD distress among the urban adult population in Saudi Arabia, using a cross-sectional study design. Data analysis was conducted using IBM SPSS software version 25 (IBM Corp., Armonk, NY, USA) and Microsoft Excel (Microsoft Corporation, Redmond, WA, USA). The study was designed with a statistical power of 80% at a cut-off value of 0.05 and 0.2. Diverse statistical approaches have been utilized to examine the connection between independent variables, including methods such as Chi-square analysis and Pearson's statistical assessment.

Result

A total of 685 respondents were included in the survey for this study, with the majority of the participants from the eastern region (31%) and between the age groups of 25 and 30 years (34%) showing a lot of enthusiasm There was a clear dominance of the Saudi population (71%) who spoke Arabic (83%). Female (65%) participants showed more interest in this survey. The collected data were organized comprehensively in tables to facilitate a better understanding of the study's findings. P-values less than 0.05 were considered significant for the obtained results.

Conclusion

The study has shown that there were a variety of depression entanglements as a result of SCD, but they were handled well with the necessary measures by medical care professionals with much-needed psychological counseling and well-equipped medical facilities.

## Introduction

Background

Sickle cell disease (SCD) is a genetic blood disorder prevalent worldwide, particularly in Saudi Arabia. The intricate interplay between physical health and psychological well-being has led to increasing interest in understanding the potential prevalence of depression among SCD patients [1]. While there is a growing body of research associating chronic illnesses with higher rates of depression, the specific context of SCD patients in Saudi Arabia remains less explored [2]. This study aims to bridge this gap by investigating the occurrence of depression among adults with SCD in Saudi Arabia.

Rationale and knowledge gap

Living with a chronic illness like SCD can impose substantial challenges on individuals, encompassing physical symptoms, medical treatments, and lifestyle adjustments [3]. This complex landscape raises the question of whether SCD patients are more susceptible to experiencing depression [4]. While some studies have hinted at a possible correlation between SCD and depression, comprehensive research tailored to the Saudi Arabian population is limited [5]. By understanding the prevalence and characteristics of depression within this unique context, healthcare professionals can develop targeted interventions to improve the overall quality of life for SCD patients [6].

Objectives

This study seeks to determine the prevalence of depression among adults with SCD in Saudi Arabia and explore potential contributing factors. By employing validated assessment tools, we aim to quantify the extent of depressive symptoms experienced by SCD patients [7]. Additionally, the study aims to identify any associations between depression and demographic variables, disease severity, treatment history, and psychosocial factors [8]. Ultimately, the findings will contribute to the existing literature by shedding light on the psychological well-being of SCD patients in Saudi Arabia and guiding future research and clinical practices to enhance the holistic care provided to this population.

## Materials and methods

Study design

This study employs a cross-sectional research design to investigate the occurrence of depression among adults with SCD in Saudi Arabia. The methodology involved the development and validation of a questionnaire, data collection, and subsequent analysis [9].

Questionnaire development

A comprehensive questionnaire was developed to assess the occurrence of depression and associated factors among SCD patients. The questionnaire was structured to capture demographic information, disease-related variables, psychosocial aspects, and validated depression assessment scales.

Translation and cultural adaptation

To ensure cultural relevance and linguistic accuracy, the questionnaire underwent a rigorous translation and adaptation process. The English questionnaire was initially translated into Arabic by bilingual experts. Subsequently, the translated version was back-translated to English by a different set of bilingual experts. Discrepancies were resolved through consensus to retain the original intent of the questions while adapting to the local cultural context.

Questionnaire validation

The translated questionnaire was subjected to a validation process to ensure its reliability and validity. This included content validation by a panel of experts in both the SCD and psychology fields. Additionally, a pilot study was conducted on a small sample of SCD patients to assess the clarity and comprehensibility of the questionnaire items. Feedback from the pilot study participants was utilized to refine the questionnaire further.

Data collection

Data collection involved recruiting a representative sample of adults diagnosed with SCD from various healthcare facilities across Saudi Arabia. Informed consent was obtained from participants before they completed the validated questionnaire. Participants were assured of the confidentiality and voluntary nature of their participation. This Institutional Human Ethics Committee-sanctioned study was conducted from April 1, 2023, to May 31, 2023, involving 685 participating volunteers. The main objective of the study was to evaluate the proportion of adults aged 18 years and older who suffer from the co-occurrence of depression and sickle cell disease, and it specifically included adults distressed by sickle cell disease.

Data analysis

The collected data underwent rigorous statistical analysis. Descriptive statistics were used to present demographic characteristics and disease-related variables. The validated depression assessment scales were analyzed to quantify the prevalence and severity of depression among SCD patients. Inferential statistics, such as Chi-square tests and regression analysis, were employed to explore relationships between depression and various factors. Data analysis was conducted using IBM SPSS software version 25 (IBM Corp., Armonk, NY, USA) and Microsoft Excel (Microsoft Corporation, Redmond, WA, USA). The study was designed with a statistical power of 80% at a cutoff value of 0.05 and 0.2. Various statistical methods were employed to investigate the relationship between independent variables, including Chi-square and Pearson's statistical analyses. Additionally, univariate and multivariate regression analyses were conducted using the Cox proportional hazard model to identify the risk factors associated with distress among adults with sickle cell disease, including those with a hereditary history of the disease [9].

Ethical considerations

The study adhered to the ethical guidelines outlined. Participant anonymity, privacy, and informed consent were upheld throughout the research. The study was conducted under the title "The Occurrence of Depression Among Adults With Sickle Cell Disease in Saudi Arabia" and was approved by the Ibn Sina National College Research Review Board Institutional Human Ethics Committee, Jeddah, Saudi Arabia, with ethical approval number IRRB-02-21052023 along with the protocol identification number 070MP15052023.

The results were tabulated in the form of tables by categorizing the questionnaires in the form of sections for easier understanding, which was well explained in the literature.

## Results

A total of 685 respondents were included in the survey for this study. Both IBM SPSS software version 25 and Microsoft Excel were used to analyze the data. The collected data have been organized comprehensively in Table 1 to facilitate a better understanding of the study's findings.

**Table 1:** The study's participation rate was measured in terms of the number of adults with SCD in Saudi Arabia who responded to the investigation's depression occurrence survey.

Survey questionnaires	Response rate: actual number (total=685)	Response rate (approximate %)
Demographic distribution		
Eastern region	212	31
Western region	206	30
Central region	116	17
Northern region	69	10
Southern region	82	12
Age group (in years)		
18-24	185	27
25-30	233	34
31-45	158	23
46-60	61	09
>60	48	07
Gender		
Male	240	35
Female	445	65
Nationality		
Saudi	487	71
Non-Saudi	198	29
Language		
Arabic	569	83
English	116	17
Educational qualification		
Bachelor's degree	219	32
Master's degree	69	10
Elementary education	212	31
Others	185	27
Occupation		
Student	103	15
Private sector employee	151	22
Public sector employee	137	20
Business	212	31
Others	82	12
Do you have sickle cell anemia?		
Yes	124	18
No	486	71
Not sure	75	11
Do you have a family history of sickle cell anemia?		
Yes	68	10
No	459	67
Not sure	158	23
Do you undergo regular blood transfusions for SCD?		
Yes	541	79
No	137	20
Not sure	7	01
Did you undergo a splenectomy for SCD?		
Yes	404	59
No	89	13
Not sure	192	28
Did you undergo a cholecystectomy for SCD?		
Yes	336	49
No	130	19
Not sure	219	32
Do you suffer from tiredness or low energy, even when rested?		
Yes	431	63
No	171	25
Not sure	83	12
Do you suffer from restlessness or difficulty concentrating?		
Yes	288	42
No	376	55
Not sure	21	3
Do you suffer from difficulty carrying out daily activities?		
Yes	322	47
No	315	46
Not sure	48	7
Do you suffer from changes in appetite or sleep patterns?		
Yes	219	32
No	466	68
Not sure	0	0
Do you suffer from aches or pains that have no obvious cause?		
Yes	377	55
No	274	40
Not sure	34	5
Do you suffer from persistent sadness, anxiousness, or irritability?		
Yes	459	67
No	191	28
Not sure	35	5
Do you suffer from a loss of interest in friends and activities that you normally enjoy?		
Yes	14	2
No	651	95
Not sure	20	03
Do you suffer from self-harming or suicidal thoughts?		
Yes	62	9
No	603	88
Not sure	20	3
Do you suffer from feelings of worthlessness, hopelessness, or guilt?		
Yes	82	12
No	295	43
Not Sure	308	45
Have you been referred for a psychiatric consultation?		
Yes	281	41
No	356	52
Not sure	48	7
Have you been diagnosed with depression?		
Yes	391	57
No	184	27
Not sure	110	16
Have you been on medication for depression?		
Yes	431	63
No	199	29
Not sure	55	08
What is the general well-being scale (on a scale of 1 to 10) of your health due to the occurrence of depression with SCD?		
1	7	1
2	15	2
3	34	5
4	27	4
5	103	15
6	15	2
7	103	15
8	68	10
9	184	27
10	129	19

The results were presented in percentage format in a table. The IBM SPSS Statistics software version 25 was employed for statistical analysis, which involved conducting a Pearson's Chi-square test to establish correlations. Results with P-values less than 0.05 were considered significant, as summarized in Table 2.

**Table 2:** P-value esteem for the study on the occurrence of depression among adults with SCD in Saudi Arabia

Survey questions	P-value esteem
Demographic distribution	0.06
Age group (in year)s	0.004
Gender	0.009
Nationality	0.07
Language	0.812
Qualification	0.007
Occupation	0.315
Do you have sickle cell anemia?	0.133
Do you have a family history of sickle cell anemia?	0.346
Do you undergo regular blood transfusions for SCD?	0.001
Did you undergo a splenectomy for SCD?	0.202
Did you undergo a cholecystectomy for SCD?	0.003
Do you suffer from tiredness or low energy, even when rested?	0.09
Do you suffer from restlessness or difficulty concentrating?	0.07
Do you suffer from difficulty carrying out daily activities?	0.002
Do you suffer from changes in appetite or sleep patterns?	0.003
Do you suffer from aches or pains that have no obvious cause?	0.325
Do you suffer from persistent sadness, anxiousness, or irritability?	0.05
Do you suffer from a loss of interest in friends and activities that you normally enjoy?	0.04
Do you suffer from self-harming or suicidal thoughts?	0.518
Do you suffer from feelings of worthlessness, hopelessness, or guilt?	0.28
Have you been referred for a psychiatric consultation?	0.38
Have you been diagnosed with depression?	0.05
Have you been on medication for depression?	0.03
What is the general well-being scale (on a scale of 1 to 10) of your health due to the occurrence of depression with SCD?	0.07

Sociodemographic data

The investigation was carried out on the adult urban population of Saudi Arabia, which included the eastern, western, central, northern, and southern regions of the country. The percentage of participants from the eastern region was 31%, the western region was 30%, the central region was 17%, the northern region was 10%, and the southern region was 12%, respectively. The analysis was obtained using Pearson's chi-square test with a P-value of 0.06.

The survey participants were asked a series of general bio-data review questions that included their nationality, age range, gender, language, education level, and occupation. The survey results showed that 71% of participants were Saudi nationals, while the remaining 29% were non-Saudis, with a statistical significance of P = 0.07. Results showed that 27% of respondents belonged to the 18-24 age group, 34% were aged between 25 and 30 years, 23% fell within the 31-45 age group, and 9% and 7% belonged to the 46-60 and over 60 age groups, respectively [10]. The results showed a significant difference for the obtained P-value of 0.004. In terms of gender distribution, around 65% of participants were female while 35% were male, with a P-value of 0.009. However, there was no significant difference in the P-value of 0.812 regarding the language choice of participants, where 83% of them selected Arabic and only 17% opted for English. The educational attainment of the respondents was analyzed, indicating that most of them held a bachelor's degree (32%), followed by those with elementary education (31%) and other educational qualifications (27%). Only 10% of the participants held a Master's degree, and the statistical analysis yielded a P-value of 0.007. Additionally, the occupational distribution of the sample was reported, with 15% of the participants being students, 22% private sector employees, 20% public sector employees, 31% businessmen, and 12% having other occupations. However, the statistical test showed that this distribution was not significant (P-value = 0.315).

Complications related to sickle cell disease

The survey questions that were posed to the public to determine their experiences with SCD were systematically organized based on the level of response obtained from participants [11]. The question "Do you have sickle cell anemia?" had 18% answering yes, 71% answering no, and 11% responding not sure. The results revealed that there was a noteworthy disparity, given the P-value of 0.133. Moreover, the survey asked participants whether they had a family history of sickle cell anemia. To this, 10% answered yes, 67% answered no, and 23% were unsure, yielding a P-value of 0.346. The question "Do you undergo regular blood transfusions for SCD?" had 79% responding yes, 20% no, and 1% not sure, with a P-value of 0.001. The survey regarding "Did you undergo splenectomy for SCD?" had 59% answering yes, 13% saying no, and 28% saying not sure, yielding a P-value of 0.202. Similarly, the poll on "Did you undergo cholecystectomy for SCD?" had a P-value of 0.003, with 49% responding yes, 19% no, and 32% not sure. The responses to the poll "Do you suffer from tiredness or low energy, even when rested?" yielded a significant P-value of 0.09, with 63% answering yes, 25% saying no, and 12% saying not sure. The survey regarding "Do you suffer from restlessness or difficulty concentrating?" showed a P-value of 0.07, with 55% responding no, 42% yes, and 3% not sure. The poll on "Do you suffer from difficulty carrying out daily activities?" had 47% Yes, 46% no, and 7% not sure, with a P-value of 0.002. The survey regarding "Do you suffer from changes in appetite or sleep patterns?" showed a P-value of 0.003, with 32% responding yes, 68% no, and 0% not sure. The final poll on "Do you suffer from aches or pains that have no obvious cause?" showed a P-value of 0.325, with 55% yes, 40% no, and 5% not sure.

The psychological complications that are associated with sickle cell disease

A set of structured survey questionnaires was developed to investigate the psychological complications associated with SCD within a specific population [12-13]. The findings were analyzed and presented as follows: Regarding the first poll, participants were asked, "Have you experienced persistent sadness, anxiousness, or irritability?". Results showed that 67% answered yes, 28% answered no, and 5% were unsure, with a significant difference evident based on the Pearson Chi-Square test (P-value = 0.05). The second survey question was targeted at determining whether participants had experienced a "loss of interest in friends or activities they normally enjoy". Only 2% of respondents answered yes, while 95% answered no, and 3% were unsure, with a P-value of 0.04. The third poll, aimed at examining "self-harming or suicidal thoughts," revealed that 9% of the participants replied yes, while 88% responded no, and 3% were unsure, with a P-value of 0.518, indicating no significant difference [14-15]. The final poll in this category, which investigated "feelings of worthlessness, hopelessness, or guilt," showed that 12% of the participants had experienced such feelings, whereas 43% answered no and 45% were unsure, with a P-value of 0.28, indicating no significant difference.

Diagnosis and prevention

A set of structured survey questionnaires was developed to investigate the diagnosis and prevention measures of sickle cell disease, as well as the level of awareness within a specific population. The first survey question targeted the population and inquired if they had been referred for a psychiatric consultation [16-17]. Findings showed that 41% responded yes, 52% responded no, and 7% were unsure. The obtained P-value of 0.38 indicates that there is no significant difference [18-19]. A similar poll was conducted to evaluate if the participants had been diagnosed with depression, with a minor variation indicating yes as the leading response, accounting for 57%, while no accounted for 27%, and 16% of the participants remained indecisive. The obtained P-value was 0.05. The final question in this category aimed to assess participants' medication intake for depression. Results showed that 63% responded yes, 29% responded no, and 8% were unsure. The P-value difference obtained in this survey was 0.03.

General well-being due to the prevalence of depression along with sickle cell disease

The ultimate overview in this cross-sectional ponder was to recognize the relationship between SCD and discouragement by inquiring members to rate their general well-being on a scale of one to 10 [20-21]. To raise awareness among the target population about the potential mental effects that SCD may have, the analysts situated this address after the survey [22]. The lion's share of the respondents (19% and 27%) appraised depression's effect on their well-being as 10 and nine individually [23]. Direct affect appraisals of five, seven, and eight earned a comparative rate of reactions of 15%, 15%, and 10%, individually [24]. The rest of the impact appraisals from one to six and nine got a rate of reactions of 1%, 2%, 5%, 4%, and 2%, respectively. The Pearson Chi-Square test made a measurably noteworthy distinction with a P-value of 0.038 for the survey category.

## Discussion

This study suggests that depression is a significant problem for adults with SCD in Saudi Arabia. The study found that a high percentage of respondents (65%) reported experiencing symptoms of depression and that these symptoms were significantly associated with several sociodemographic factors, including age, gender, and educational level [18]. The study also found that depression was significantly associated with several medical factors, including the severity of SCD, the number of hospital visits, and the use of pain medication [25].

The findings of this study highlight the importance of screening for depression in adults with SCD. Early identification and treatment of depression can help to improve the quality of life for people with SCD and reduce the risk of serious complications [26]. The study also suggests that there is a need for more research into the causes and treatment of depression in people with SCD.

The major finding of this pivotal research study was that depression is a significant problem for adults with SCD in Saudi Arabia. The prevalence of depression is higher in adults with SCD than in the general population [5]. Depression is associated with several sociodemographic factors, including age, gender, and educational level. Depression is also associated with several medical factors, including the severity of SCD, the number of hospital visits, and the use of pain medication. Early identification and treatment of depression can help improve the quality of life for people with SCD. There is a need for more research into the causes and treatment of depression in people with SCD. The findings of this study have important implications for the care of adults with SCD in Saudi Arabia [8-9]. The high prevalence of depression in this population suggests that screening for depression should be a routine part of care. Early identification and treatment of depression can help to improve the quality of life for people with SCD and reduce the risk of serious complications [27].

Depression is also associated with several medical factors, including the severity of SCD, the number of hospital visits, and the use of pain medication [11]. The study found that depression was more common in adults with SCD who had a more severe disease, had more frequent hospital visits, and used more pain medication [3]. This suggests that the physical and emotional challenges of living with SCD can contribute to the development of depression.

Early identification and treatment of depression can help improve the quality of life for people with SCD. The study found that adults with SCD who received treatment for depression reported significant improvements in their quality of life. This suggests that early identification and treatment of depression can be an important part of improving the lives of people with SCD [28].

There is a need for more research into the causes and treatment of depression in people with SCD. The study found that there is still much that we do not know about the causes and treatment of depression in people with SCD [29]. More research is needed to better understand these factors so that we can develop more effective interventions [3].

Overall, the findings of this study suggest that depression is a significant problem for adults with SCD in Saudi Arabia. The high prevalence of depression in this population highlights the importance of screening for depression and providing treatment to those who need it [15-16]. More research is needed to better understand the causes and treatment of depression in people with SCD so that we can develop more effective interventions [30].

Strengths of the study

The research offers significant contributions by addressing the prevalence of depression among adults with sickle cell disease (SCD) in Saudi Arabia. With a substantial sample size of 685 participants from diverse regions, the study provides a comprehensive understanding. Administering the survey in both Arabic and English enhances accessibility, and the inclusion of various factors like demographic data, complications, and psychological well-being offers a holistic view. Rigorous statistical analyses were conducted to establish relationships between variables. Ethical approval highlights the study's adherence to ethical standards, and the results contribute to raising awareness about the importance of medical and psychological care for this population.

Weaknesses of the study

However, the research possesses certain limitations. An online survey introduces potential sampling bias, excluding those without internet access. Self-reported data could be influenced by recall or social desirability bias, impacting accuracy. Its cross-sectional design limits causal inference. Generalizability is restricted due to the study's focus on Saudi Arabia. The diagnosis of depression relied solely on self-reported symptoms rather than clinical assessment. Insufficient socioeconomic exploration overlooks potential impacts. Non-response bias might be present, skewing participant representation. The study lacks in-depth intervention strategies for addressing depression. Transparent conflict of interest disclosure is needed. Publication bias and the absence of longitudinal data limit comprehensive insights into depression progression among SCD adults.

## Conclusions

This study employs a systematic approach to investigate the occurrence of depression among adults with sickle cell disease in Saudi Arabia. By rigorously developing, translating, validating, and administering the questionnaire, we aim to generate reliable insights into the psychological well-being of this population, facilitating targeted interventions and improved patient care. The findings of the study suggest that screening for depression and providing treatment to those who need it should be a priority in the care of adults with SCD in Saudi Arabia. This cross-sectional study was a small effort in order to lead such important examinations in preventive medicine.
